# Procalcitonin as a biomarker for predicting bacterial infection in chimeric antigen receptor T‐cell therapy recipients

**DOI:** 10.1002/cam4.5665

**Published:** 2023-02-08

**Authors:** Marissa Z. Powell, Kristin C. Mara, Radhika Bansal, Matthew A. Hathcock, Arushi Khurana, N. Nora Bennani, Yucai Wang, Jonas Paludo, Jose Villasboas Bisneto, Stephen M. Ansell, Patrick B. Johnston, Yi Lin, Jason N. Barreto

**Affiliations:** ^1^ Department of Pharmacy Mayo Clinic Rochester Minnesota USA; ^2^ Department of Quantitative Health Sciences Mayo Clinic Rochester Minnesota USA; ^3^ Division of Hematology, Department of Internal Medicine Mayo Clinic Rochester Minnesota USA

**Keywords:** antibiotics, antimicrobial stewardship, chimeric antigen receptor, cytokine release syndrome, fever, immunotherapy, infection, procalcitonin, T‐cell

## Abstract

**Background:**

It is unknown whether serum procalcitonin (PCT) concentration monitoring can differentiate between bacterial infection or cytokine release syndrome (CRS) when chimeric antigen receptor T‐cell (CAR‐T) recipients present with a constellation of signs and symptoms that may represent both complications.

**Objective:**

The objective of the study was to assess the utility of serum PCT concentrations as a biomarker of bacterial infection in CAR‐T recipients.

**Study design:**

This single‐center, retrospective, medical record review evaluated patients prescribed CAR‐T therapy until death or 30 days after infusion. Logistic regression modeling determined the association between elevated serum PCT concentrations within 48 h of fever onset and microbiologically confirmed infection. Secondary outcomes included clinically suspected infection, CAR‐T toxicity rates, and broad‐spectrum antibiotic usage. Predictive performance of PCT was assessed by area under the receiver operating characteristic curve (AUC).

**Results:**

The 98 included patients were a median age of 63 (IQR: 55–69) years old, 47 (48%) were male, and 87 (89%) were Caucasian. Baseline demographics and clinical characteristics were similar between patients with and without a bacterial infection. Serum PCT >0.4 ng/mL within 48 h of fever was significantly associated with a microbiologically confirmed bacterial infection (OR: 2.75 [95% CI: 1.02–7.39], *p* = 0.045). Median PCT values in patients with and without confirmed infections were 0.40 ng/mL (IQR: 0.26, 0.74) and 0.26 ng/mL (IQR: 0.13, 0.47), respectively. The AUC for PCT to predict bacterial infection was 0.62 (95% CI 0.48–0.76). All patients experienced CRS of some grade, with no difference in CRS severity based on elevated PCT. Broad‐spectrum antibiotics were used for a median of 45% and 23% of days in those with and without confirmed infection, respectively (*p* = 0.075).

**Conclusion:**

Elevated serum PCT concentrations above 0.4 ng/mL at time of first fever after CAR‐T infusion was significantly associated with confirmed bacterial infection. Furthermore, rigorous, prospective studies should validate our findings and evaluate serial PCT measurements to optimize antimicrobial use after CAR‐T therapy.

## Graphical abstract

A serum procalcitonin concentration >0.4 ng/mL was significantly associated with confirmed bacterial infection in patients after receiving chimeric antigen T‐cell therapy. Procalcitonin may be a useful marker for differentiating bacterial infection from cytokine release syndrome in chimeric antigen T‐cell therapy recipients.

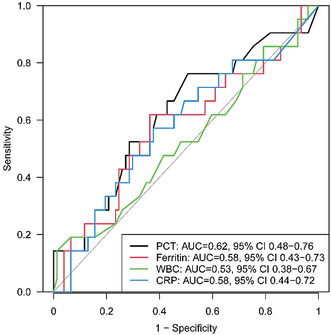

## 1 INTRODUCTION

Chimeric antigen receptor T‐cell (CAR‐T) therapy represents a major advancement in the management of refractory or relapsed hematologic malignancies.^1^, ^2^, ^3^, ^4^ CAR‐T therapy is not without toxicities and clinical complexity following administration.^5^, ^6^, ^7^, ^8^, ^9^ Cytokine release syndrome (CRS) is a common toxicity following CAR‐T therapy that is characterized by high fevers, tachycardia, hypotension, and hypoxia.^5^

Bacterial infection is another well‐known complication, with particularly high risk during the first 30 days following CAR‐T infusion.^6^ Bacterial infection and CRS have similar presentations but vastly different management strategies.^10^, ^11^, ^12^, ^13^, ^14^ The inability to differentiate between CRS and bacterial infection can lead to unnecessary antibiotic administration. The negative consequences of unnecessary antibiotic prescribing include increased hospital length of stay, increased hospital costs, potential antibiotic‐associated *Clostridium difficile*, secondary infections, and emergence of multidrug‐resistant bacteria.^15^

Procalcitonin (PCT) is a calcitonin precursor hormone that is upregulated in response to circulating bacteria or endotoxins.^16^ Studies have demonstrated that serum PCT concentration monitoring has utility in the diagnosis and management of sepsis and lower respiratory tract infection.^18^, ^19^, ^20^, ^21^, ^22^, ^23^ Typically, PCT levels begin rising within 3–4 h of bacterial infection and usually peak between 12 and 36 h.^17^ Additionally, serum PCT monitoring has been found to successfully predict infection, particularly bacteremia, in patients with hematological malignancies.^24^, ^25^, ^26^, ^27^, ^28^ Lastly, longitudinal serum PCT monitoring can facilitate antibiotic discontinuation without compromising patient safety.^29^, ^30^

It is unknown whether serum PCT concentration monitoring can differentiate between bacterial infection or CRS when CAR‐T therapy recipients present with a constellation of signs and symptoms that represent either complication. This study sought to evaluate whether serum PCT concentrations could indicate microbiologically confirmed bacterial infection and predict any bacterial infection.

## 2 MATERIALS AND METHODS

This Mayo IRB‐approved single‐center, retrospective, medical record review evaluated consecutive adult patients diagnosed with B‐cell malignancy who received CAR‐T therapy between 2018 and 2021 until 30 days after the CAR‐T infusion or death. Patients who did not experience a fever after CAR‐T infusion or have a serum PCT level within 48 hours of fever onset were excluded.

The primary outcome was the association between elevated serum PCT concentrations within 48 h of fever onset and microbiologically confirmed bacterial infection. Secondary outcomes included the association between elevated PCT and incidence of clinically suspected infection, a comparison of CRS rate and severity in those with and without bacterial infection, and frequency of broad‐spectrum antibiotic use. Clinically suspected infection was determined by author discretion based on symptomatic presentation and radiographic evidence of infection. CRS was graded according to the American Society for Blood and Marrow Transplantation (ASTCT) consensus guidelines.^5^ In addition to serum PCT; ferritin, white blood cell count (WBC), and C‐reactive protein (CRP) were assessed as distinguishing laboratory markers indicative of CRS or bacterial infection.

Frequencies and percentages summarized categorical data, and medians and interquartile ranges summarized continuous data. Baseline characteristics were compared between those who did and did not have a bacterial infection using either a chi‐squared for Fisher's exact test for categorical data, and the Wilcoxon rank sum test for continuous data. The association between PCT levels and bacterial infection was assessed using logistic regression. Associations were summarized using odds ratios (OR) and 95% confidence intervals (CI). A cut‐point analysis was done to determine the optimal cutoff for PCT to distinguish who would be considered high risk for infection. The sensitivity, specificity, positive predictive value (PPV) and negative predictive value (NPV) for the new cutoff were assessed. The discrimination of each biomarker was evaluated using the area under the receiver operating characteristic curve (AUC). All analyses were performed using SAS version 9.4 software (SAS Institute Inc.).

## 3 RESULTS

The 98 included patients were a median age of 62.7 (IQR: 54.7–69.0) years, 47 (48%) were male and 87 (88.8%) were Caucasian. Baseline demographics and clinical characteristics were similar between groups except for a lower alkaline phosphate in patients without confirmed bacterial infection (Table 1). The median (IQR) time to first fever of 4 (2, 6) days from CAR‐T cell infusion date into the result section. We have also added the median (IQR) number of 10 (7, 12) cultures drawn as part of the infectious workup during empiric broad‐spectrum antibiotic therapy.

**TABLE 1:** Association of baseline demographics and laboratory values with confirmed bacterial infection.

	No bacterial infection (*N* = 77)	Bacterial infection (*N* = 21)	Total (*N* = 98)	*p* value
Age, median (IQR)	63.6 (54.7, 69.9)	60.1 (57.3, 65.7)	62.7 (54.7, 69.0)	0.21
Male, *n* (%)	39 (50.6)	8 (38.1)	47 (48.0)	0.31
Race/Ethnicity, *n* (%)				
White	67 (87.0)	20 (95.2)	87 (88.8)	0.62
American Indian or Alaska Native	1 (1.3)	1 (4.8)	2 (2.0)	
Asian	2 (2.6)	0 (0.0)	2 (2.0)	
Black or African American	2 (2.6)	0 (0.0)	2 (2.0)	
Other	3 (3.9)	0 (0.0)	3 (3.1)	
Unknown	2 (2.6)	0 (0.0)	2 (2.0)	
Ethnicity, *n* (%)				
Hispanic or Latino	2 (2.6)	0 (0.0)	2 (2.0)	0.047
Not Hispanic or Latino	72 (93.5)	17 (81.0)	89 (90.8)	
Unknown	3 (3.9)	4 (19.0)	7 (7.1)	
Diagnosis subgroup, *n* (%)				
Lymphoma	48 (62.3)	18 (85.7)	66 (67.3)	0.30
Multiple myeloma	27 (35.1)	3 (14.3)	30 (30.6)	
Other	2 (2.6)	0	2 (2)	
Prior SCT, *n* (%)	44 (57.1)	11 (52.4)	55 (56.1)	0.70
Hemoglobin, median (IQR)	9.3 (8.5, 10.6)	8.9 (8.4, 10.0)	9.2 (8.5, 10.6)	0.41
Platelets, median (IQR)	114 (56, 155)	148 (97, 206)	117 (60, 172)	0.13
White blood cell count, median (IQR)	1.5 (0.7, 2.2)	1.3 (0.7, 2.1)	1.5 (0.7, 2.2)	0.73
Absolute neutrophil count, median (IQR)	1.38 (0.64, 1.97)	1.10 (0.73, 2.06)	1.26 (0.67, 2.06)	0.85
Absolute lymphocyte count, median (IQR)	0.06 (0.03, 0.60)	0.34 (0.03, 0.71)	0.08 (0.03, 0.60)	0.47
BUN, median (IQR)	13 (11, 16)	15 (13, 18)	13 (11, 17)	0.22
Serum creatinine, median (IQR)	0.84 (0.72, 1.02)	0.78 (0.68, 0.90)	0.81 (0.72, 1.00)	0.16
Total bilirubin, median (IQR)	0.5 (0.3, 0.5)	0.4 (0.3, 0.6)	0.5 (0.3, 0.5)	0.63
Direct bilirubin^a^, median (IQR)	0.2 (0.2, 0.2)	0.2 (0.2, 0.2)	0.2 (0.2, 0.2)	0.48
ALT, median (IQR)	20 (15, 32)	18 (12, 29)	20 (14, 32)	0.29
AST, median (IQR)	26 (21, 38)	26 (23, 32)	26 (21, 37)	0.89
Alkaline phosphatase, median (IQR)	79 (64, 100)	96 (81, 127)	83 (68, 107)	0.039
Albumin, median (IQR)	3.8 (3.5, 4.2)	3.9 (3.3, 4.1)	3.9 (3.4, 4.2)	0.53
Lactate dehydrogenase^b^, median (IQR)	240 (197, 356)	312 (220, 521)	242 (204, 406)	0.12
CD4 count^c^, median (IQR)	62 (14, 175)	232 (89, 281)	80 (14, 230)	0.017
CD4 percent^c^, median (IQR)	38 (23, 60)	45 (31, 64)	38 (26, 60)	0.22
Ferritin, median (IQR)	374 (169, 1122)	785 (198, 1419)	384 (169, 1395)	0.28
CRP, median (IQR)	11.1 (4.4, 29.7)	16.7 (7.6, 54.7)	11.6 (4.4, 41.9)	0.27
Immunoglobulin G^a^, median (IQR)	507 (321, 801)	467 (310, 596)	487 (310, 760)	0.49

^a^ Available in 94 (73 with no infection, 21 with an infection).

^b^ Available in 63 (48 with no infection, 15 with an infection).

^c^ Available in 90 (70 with no infection, 20 with an infection).

**FIGURE 1:**
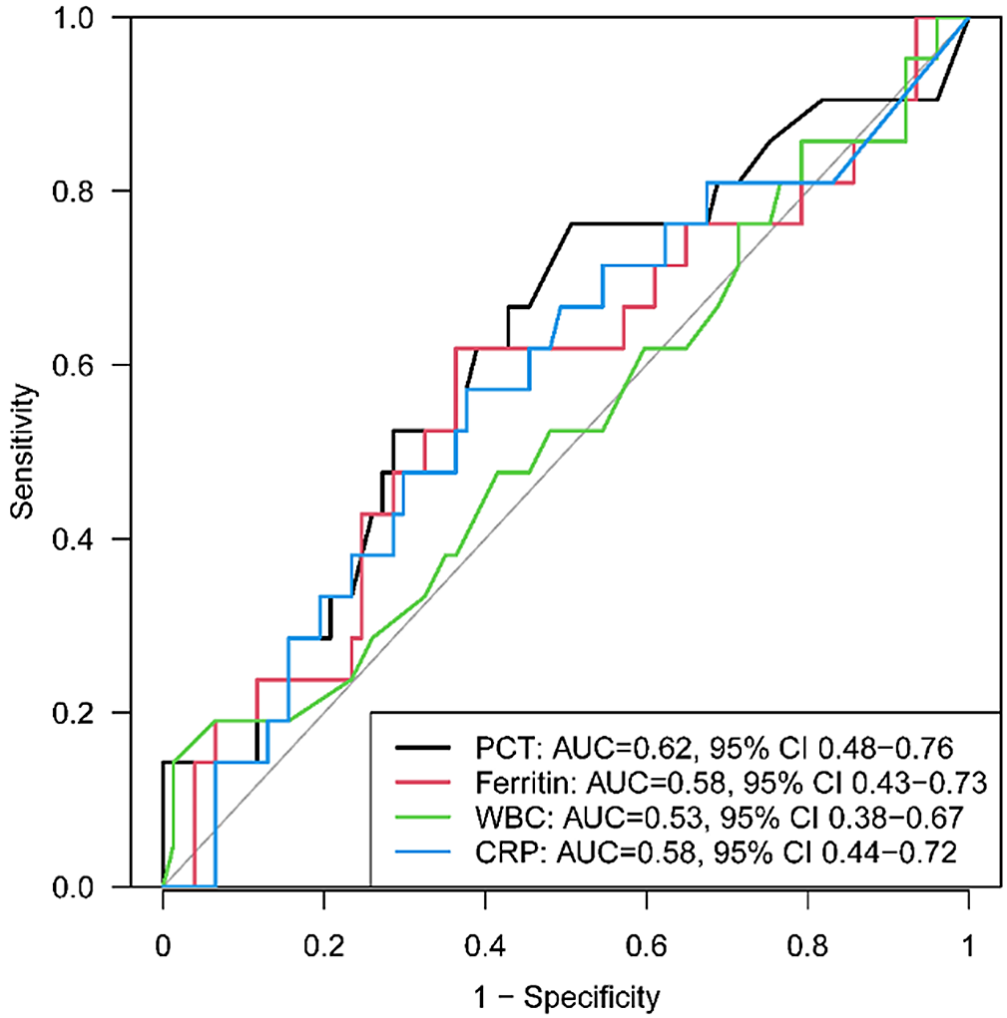
Diagnostic performance of procalcitonin, ferritin, white blood cell count, and C‐reactive protein in predicting bacterial infection.

Median PCT values in patients with and without confirmed bacterial infections were 0.40 ng/mL (IQR: 0.26, 0.74) and 0.26 ng/mL (IQR: 0.13, 0.47), respectively. Serum PCT ≥0.4 ng/mL within 48 h of fever was significantly associated with the likelihood of a microbiologically confirmed bacterial infection (OR: 2.75 [95% CI: 1.02–7.39], *p* = 0.045). Other bacterial infection outcomes are detailed in Table 2. PCT with a cutoff value of 0.4 ng/mL carried a sensitivity of 0.52 (95% CI 0.30–0.74), specificity of 0.71 (95% CI 0.60–0.81), PPV of 0.33 (95% CI 0.18–0.52) and a NPV of 0.85 (95% CI 0.74–0.92). With an AUC of 0.62 (95% CI 0.48–0.76), PCT demonstrated similar performance to ferritin, WBC, and CRP in predicting bacterial infection (Figure 1). We did not find statistically significant cutoffs for WBC or CRP and detection parameters of WBC count and CRP are provided in Table 2.

**TABLE 2:** Test Characteristics for procalcitonin, white blood cell count, and C‐reactive protein cutoffs.

Parameter	OR (95% CI)	*p*‐value	AUC	Sensitivity	Specificity	PPV	NPV
Procalcitonin ≥0.4	2.75 (1.02–7.39)	0.045	0.62	0.52	0.71	0.33	0.85
WBC <0.4	3.39 (0.82–13.98)	0.09	0.56	0.19	0.94	0.44	0.81
CRP ≥15.7	2.21 (0.83–5.88)	0.11	0.60	0.57	0.62	0.29	0.84

All patients experienced CRS of some grade, with no statistically significant difference in CRS severity based on elevated PCT (Table 3). Broad‐spectrum antibiotics were used for a median of 33% and 23% of days in patients with and without confirmed bacterial infection, respectively (*p* = 0.33). The most common sites of microbiologically confirmed bacterial infections were central venous catheters (*n* = 11), urine (*n* = 4), and blood (*n* = 3). Organisms grown from culture were mostly of the *Staphylococcus* genus (*n* = 18), including *Staphylococcus epidermidis* (*n* = 10), *Staphylococcus hominis* (*n* = 2), and *Staphylococcus haemolyticus* (*n* = 2), *Staphylococcus aureus* (*n* = 1) and coagulase‐negative *Staphylococcus* species not otherwise specified (*n* = 3). Other organisms detected during infectious work‐up included *Clostridium difficile* (*n* = 2), *Escherichia coli* (*n* = 1), *Klebsiella pneumoniae* (*n* = 1), *Terrisporobacter glycolicus* (*n* = 1), and *Micrococcus luteus* (*n* = 1). Clinically suspected infections were primarily pneumonia (*n* = 16), intra‐abdominal infections (*n* = 3), or cellulitis (*n* = 2). There were zero invasive fungal infections during infection workup. Additionally, there were 14 positive viral cultures including one patient with a positive herpes simplex virus swab from the mouth, 6 patients with viral organisms detected in respiratory secretions, and one patient with BK virus positive in the urine. Other viral cultures included transient detection of cytomegalovirus by polymerase chain reaction during routine surveillance blood cultures.

**TABLE 3:** Association of serum procalcitonin with bacterial infection and toxicity outcomes.

Endpoints evaluated	Serum procalcitonin ≥0.4 ng/mL (*n* = 33)	Serum procalcitoni*n* <0.4 ng/mL (*n* = 65)	Odds ratio (95% confidence interval)	*p*‐value
Bacterial infection outcomes				
Microbiologically confirmed infections, *N* (%)	11 (33.3)	10 (15.4)	2.75 (1.02–7.39)	0.045
Clinically suspected infection, *N* (%)	9 (27.3)	12 (18.5)	1.66 (0.62–4.46)	0.32
Microbiologically confirmed or clinically suspected infection, *N* (%)	16 (48.5)	19 (29.2)	2.28 (0.96–5.42)	0.063
Toxicity outcomes				
CRS maximum grade, *N* (%)				
1	13 (39.4)	34 (52.3)	Reference	
2	17 (51.5)	29 (44.6)	1.52 (0.63–3.63)	0.35
3	3 (9.1)	1 (1.5)	5.96 (0.64–55.51)	0.12
4	0 (0.0)	1 (1.5)	0.77 (0.01–83.70)	0.91
Severe CRS (3/4)	3 (9.1)	2 (3.1)	3.15 (0.50–19.86)	0.22
CRS management medication usage, *N* (%)				
None	11 (33.3)	31 (47.7)	Reference	
Steroids	1 (3.0)	3 (4.6)	0.94 (0.09–10.00)	0.96
Tocilizumab	2 (6.1)	4 (6.2)	1.41 (0.23–8.80)	0.71
Tocilizumab and steroids	19 (57.6)	27 (41.5)	1.98 (0.80–4.90)	0.14
Neurotoxicity, *N* (%)	17 (51.5)	27 (41.5)	1.50 (0.64–3.47)	0.35

## 4 DISCUSSION

Our study demonstrated that a serum PCT ≥0.4 ng/mL within 48 h of fever in patients who have received CAR‐T cell therapy were significantly more likely to have a confirmed bacterial infection compared to those with a serum PCT <0.4 ng/mL. PCT with a cutoff value of 0.4 ng/mL had fair test characteristics; however, clinical relevance was variable with a low PPV and a fair NPV. The predictive performance of PCT for infection was similar to that of ferritin, WBC, and CRP.

Procalcitonin has been studied as a predictor of bacterial infection in many clinical environments and different patient populations and its utility remains controversial.^31^, ^32^, ^33^ Previously determined PCT thresholds, diagnostic accuracy, and predictive performance have varied by immune status, disease state, patient population, clinical presentation, outcome studied, and test used.^16^, ^19^, ^20^, ^25^, ^26^, ^27^, ^28^, ^34^, ^35^ The cutoff of 0.4 ng/mL determined in our study differs from the 0.25 ng/mL that is commonly used for community‐acquired bacterial pneumonia.^21^ Additionally, it is lower than the cutoff value of 1.5 ng/mL that demonstrated utility in diagnosing bacterial infections in patients undergoing hemodialysis.^36^ However, previous studies have suggested that differences of PCT concentrations exist in patients with hematologic malignancies.^24^, ^25^, ^27^, ^28^, ^34^, ^37^, ^38^, ^39^ Interestingly, our PCT value was similar to another study that assessed this biomarker in CAR‐T therapy recipients.^40^

Differences in study design and outcomes preclude direct comparison; however, one reason that PCT was likely not associated with infection in some other evaluations was the inclusion of viral, fungal, and other infection types; whereas, our outcome focused solely on any bacterial infection, regardless of severity. The increased incidence of Gram‐positive organisms as the causative pathogen in our study is likely due to fluoroquinolones prescribed as antibacterial prophylaxis during the period of neutropenia as part of our institutional protocol in accordance with published recommendations.^11^ Studies have demonstrated serum PCT concentrations in patients with a bacterial infection caused by a Gram‐positive organism are generally lower than in patients with infections and sepsis due to Gram‐negative organisms.^35^, ^41^ Furthermore, serum PCT has the ability to distinguish blood contamination from bloodstream infection due to coagulase‐negative staphylococci.^26^ This is important because these organisms may have clinical significance, particularly when detected in an immunocompromised host.^42^, ^43^

The predictive performance of PCT was poor for use as a solitary, independent, diagnostic marker; though, expert consensus recommends it as an adjunct assay in combination with clinical and microbiological assessment.^44^, ^45^, ^46^ This result is below what has been found in other studies assessing PCT in patients with malignancy and might reflect the difference in inflammation experienced by patients diagnosed with CRS after CAR T‐cell infusion. However, the predictive performance was similar to that of other markers commonly used in clinical practice to support a diagnosis of infection. We feel that the overlap between both clinical and chemical presentations of bacterial infection and CRS is what rendered WBC count and CRP inconsequential for a differential diagnosis specific for infection. Cytopenias are common after lymphodepletion and can be profound and prolonged in up to 46% of CAR‐T recipients.^47^ Additionally, marked elevations in C‐reactive protein and ferritin can occur during CRS is not specific for CRS or infection, but is often used to follow inflammation that can result from either diagnosis.^5^

Several guidelines recommend utilizing PCT levels plus clinical criteria to guide antibiotic discontinuation.^40^, ^48^ CAR‐T recipients experience high antimicrobial usage with current recommendations favoring antimicrobial prophylaxis.^11^, ^14^, ^49^ Additionally, the high incidence of CRS in CAR‐T recipients that is indistinguishable from infection when presenting with fevers during neutropenia compels prompt administration of appropriate broad‐spectrum antibacterial therapy.^10^, ^11^, ^14^ This is evident in our study where broad‐spectrum antibiotics were used for a median 23% of hospital days in patients without confirmed infection. This indicates a need for better stewardship that can prevent or minimize antibiotic overuse in this vulnerable patient population to abrogate the many negative consequences that could follow.^50^

Limitations include the small sample size, retrospective design, and unblinded nature of data collection. Additionally, baseline PCT values were missing in a substantial amount of our patients. This information should be collected in future studies, though a study in a similar patient population showed a median PCT at baseline that was nearly undetectable, but with a variable range.^40^ Third, our study included patients with three different baseline cancer diagnoses and evaluating serum PCT performance independently in patients with lymphoma and multiple myeloma may reveal disease‐specific parameters. Fourth, the heterogeneity of our confirmed infections may have impacted performance and focusing on a particular site of infection could demonstrate greater utility in serum PCT measurements. Lastly, PCT values trended over time were also unavailable in the majority of patients, precluding our ability to understand the utility of PCT in assisting with antibiotic de‐escalation or discontinuation.

## 5 CONCLUSION

Our study demonstrated a significant association between a serum PCT ≥0.4 ng/mL within 48 h of fever and a microbiologically confirmed bacterial infection in CAR‐T recipients. Large, prospective investigations are needed to confirm our findings and evaluate a PCT‐guided strategy for appropriate initiation and discontinuation of antibiotics in CAR‐T therapy recipients to optimize clinical and stewardship outcomes.

## AUTHOR CONTRIBUTIONS

**Marissa Z. Powell:** Conceptualization (lead); data curation (lead); formal analysis (equal); methodology (lead); writing – original draft (lead); writing – review and editing (lead). **Kristin Mara:** Conceptualization (equal); data curation (equal); formal analysis (lead); methodology (lead); writing – original draft (equal); writing – review and editing (equal). **Radhika Bansal:** Conceptualization (equal); data curation (equal); formal analysis (equal); methodology (equal); writing – original draft (equal); writing – review and editing (equal). **Matthew A. Hathcock:** Conceptualization (equal); data curation (equal); formal analysis (equal); methodology (equal); writing – original draft (equal); writing – review and editing (equal). **Arushi Khurana:** Conceptualization (equal); formal analysis (equal); methodology (equal); writing – original draft (equal); writing – review and editing (equal). **N. Nora Bennani:** Conceptualization (equal); formal analysis (equal); methodology (equal); writing – original draft (equal); writing – review and editing (equal). **Yucai Wang:** Formal analysis (equal); methodology (equal); writing – original draft (equal); writing – review and editing (equal). **Jonas Paludo:** Conceptualization (equal); formal analysis (equal); methodology (equal); writing – original draft (equal); writing – review and editing (equal). **JV Villasboas:** Conceptualization (equal); formal analysis (equal); methodology (equal); writing – original draft (equal); writing – review and editing (equal). **Stephen Ansell:** Conceptualization (equal); formal analysis (equal); methodology (equal); writing – original draft (equal); writing – review and editing (equal). **Patrick B Johnston:** Conceptualization (equal); formal analysis (equal); methodology (equal); writing – original draft (equal); writing – review and editing (equal). **Yi Lin:** Conceptualization (equal); data curation (equal); formal analysis (equal); methodology (equal); resources (lead); writing – original draft (equal); writing – review and editing (equal). **Jason n. Barreto:** Conceptualization (equal); data curation (equal); formal analysis (equal); methodology (equal); resources (equal); writing – original draft (equal); writing – review and editing (equal).

## CONFLICT OF INTEREST STATEMENT

Dr. N. Nora Bennani has participated on advisory boards for Purdue Pharma, Verastem Oncology, Acrotech Biopharma, and Sea Gen, Inc. Dr. Yi Lin has served as a consultant or received research funding from Kite/Gilead, Celgene, JUNO, Bluebird Bio, Janssen, Legend BioTech, Gamida Cells, Novartis, Merck, Takeda. Has participated on an advisory board for Sorrento: Data and Safety Monitoring Board (DSMB).

## Data Availability

The deidentified data supporting this study's findings are available on request from the corresponding author. The data are not publicly available due to privacy or ethical restrictions.

## References

[1] Neelapu SS , Locke FL , Bartlett NL , et al. Axicabtagene ciloleucel CAR T‐cell therapy in refractory large B‐cell lymphoma. N Engl J Med. 2017;377(26):2531‐2544. 29226797 10.1056/NEJMoa1707447 PMC5882485

[2] Schuster SJ , Bishop MR , Tam CS , et al. Tisagenlecleucel in adult relapsed or refractory diffuse large B‐cell lymphoma. N Engl J Med. 2019;380(1):45‐56. 30501490 10.1056/NEJMoa1804980

[3] Wang M , Munoz J , Goy A , et al. KTE‐X19 CAR T‐cell therapy in relapsed or refractory mantle‐cell lymphoma. N Engl J Med. 2020;382(14):1331‐1342. 32242358 10.1056/NEJMoa1914347 PMC7731441

[4] Raje N , Berdeja J , Lin Y , et al. Anti‐BCMA CAR T‐cell therapy bb2121 in relapsed or refractory multiple myeloma. N Engl J Med. 2019;380(18):1726‐1737. 31042825 10.1056/NEJMoa1817226 PMC8202968

[5] Lee DW , Santomasso BD , Locke FL , et al. ASTCT consensus grading for cytokine release syndrome and neurologic toxicity associated with immune effector cells. Biol Blood Marrow Transplant. 2019;25(4):625‐638. 30592986 10.1016/j.bbmt.2018.12.758 PMC12180426

[6] Zhu F , Wei G , Liu Y , et al. Incidence and risk factors associated with infection after chimeric antigen receptor T cell therapy for relapsed/refractory B‐cell malignancies. Cell Transplant. 2021;30:9636897211025503. 34144648 10.1177/09636897211025503 PMC8216343

[7] Hill JA , Li D , Hay KA , et al. Infectious complications of CD19‐targeted chimeric antigen receptor‐modified T‐cell immunotherapy. Blood. 2018;131(1):121‐130. 29038338 10.1182/blood-2017-07-793760 PMC5755046

[8] Kambhampati S , Sheng Y , Huang CY , et al. Infectious complications in relapsed refractory multiple myeloma patients after BCMA car t‐cell therapy. Blood Adv. 2022;6(7):2045‐2054. 10.1182/bloodadvances.2020004079 34543400 PMC9006279

[9] Baird JH , Epstein DJ , Tamaresis JS , et al. Immune reconstitution and infectious complications following axicabtagene ciloleucel therapy for large B‐cell lymphoma. Blood Adv. 2021;5(1):143‐155. 33570626 10.1182/bloodadvances.2020002732 PMC7805341

[10] Freifeld AG , Bow EJ , Sepkowitz KA , et al. Clinical practice guideline for the use of antimicrobial agents in neutropenic patients with cancer: 2010 update by the Infectious Diseases Society of America. Clin Infect Dis. 2011;52(4):427‐431. 21205990 10.1093/cid/ciq147

[11] Jain T , Bar M , Kansagra AJ , et al. Use of chimeric antigen receptor T cell therapy in clinical practice for relapsed/refractory aggressive B cell non‐Hodgkin lymphoma: an expert panel opinion from the American Society for Transplantation and Cellular Therapy. Biol Blood Marrow Transplant. 2019;25(12):2305‐2321. 31446199 10.1016/j.bbmt.2019.08.015

[12] Hayden PJ , Roddie C , Bader P , et al. Management of adults and children receiving CAR T‐cell therapy: 2021 best practice recommendations of the European Society for Blood and Marrow Transplantation (EBMT) and the Joint Accreditation Committee of ISCT and EBMT (JACIE) and the European Haematology association (EHA). Ann Oncol. 2022;33(3):259‐275. 34923107 10.1016/j.annonc.2021.12.003

[13] Kansagra AJ , Frey NV , Bar M , et al. Clinical utilization of chimeric antigen receptor T‐cells (CAR‐T) in B‐cell acute lymphoblastic leukemia (ALL)‐an expert opinion from the European Society for Blood and Marrow Transplantation (EBMT) and the American Society for Blood and Marrow Transplantation (ASBMT). Bone Marrow Transplant. 2019;54(11):1868‐1880. 31092900 10.1038/s41409-019-0451-2 PMC8268756

[14] Yakoub‐Agha I , Chabannon C , Bader P , et al. Management of adults and children undergoing chimeric antigen receptor T‐cell therapy: best practice recommendations of the European Society for Blood and Marrow Transplantation (EBMT) and the joint accreditation committee of ISCT and EBMT (JACIE). Haematologica. 2020;105(2):297‐316. 31753925 10.3324/haematol.2019.229781 PMC7012497

[15] Dellit TH , Owens RC , McGowan JE Jr , et al. Infectious Diseases Society of America and the Society for Healthcare Epidemiology of America guidelines for developing an institutional program to enhance antimicrobial stewardship. Clin Infect Dis. 2007;44(2):159‐177. 17173212 10.1086/510393

[16] Dandona P , Nix D , Wilson MF , et al. Procalcitonin increase after endotoxin injection in normal subjects. J Clin Endocrinol Metab. 1994;79(6):1605‐1608. 7989463 10.1210/jcem.79.6.7989463

[17] Covington EW , Roberts MZ , Dong J . Procalcitonin monitoring as a guide for antimicrobial therapy: a review of current literature. Pharmacotherapy. 2018;38(5):569‐581. 29604109 10.1002/phar.2112

[18] Commissioner Oot . FDA clears test to help manage antibiotic treatment for lower respiratory tract infections and sepsis. Accessed June 23, 2020. https://wwwfdagov/news‐events/press‐announcements/fda‐clears‐test‐help‐manage‐antibiotic‐treatment‐lower‐respiratory‐tract‐infections‐and‐sepsis#:~:text=FDA%20clears%20test%20to%20help,tract%20infections%20and%20sepsis%20%7C%20FDA

[19] Schuetz P , Bretscher C , Bernasconi L , Mueller B . Overview of procalcitonin assays and procalcitonin‐guided protocols for the management of patients with infections and sepsis. Expert Rev Mol Diagn. 2017;17(6):593‐601. 28443360 10.1080/14737159.2017.1324299

[20] Riedel S . Procalcitonin and the role of biomarkers in the diagnosis and management of sepsis. Diagn Microbiol Infect Dis. 2012;73(3):221‐227. 22704255 10.1016/j.diagmicrobio.2012.05.002

[21] Metlay JP , Waterer GW , Long AC , et al. Diagnosis and treatment of adults with community‐acquired pneumonia. An Official Clinical Practice Guideline of the American Thoracic Society and Infectious Diseases Society of America. Am J Respir Crit Care Med. 2019;200(7):e45‐e67. 31573350 10.1164/rccm.201908-1581ST PMC6812437

[22] Commissioner Oot . FDA clears test to help manage antibiotic treatment for lower respiratory tract infections and sepsis. FDA 2020.

[23] Huang DT , Yealy DM , Filbin MR , et al. Procalcitonin‐guided use of antibiotics for lower respiratory tract infection. N Engl J Med. 2018;379(3):236‐249. 29781385 10.1056/NEJMoa1802670 PMC6197800

[24] Yang M , Choi SJ , Lee J , et al. Serum procalcitonin as an independent diagnostic markers of bacteremia in febrile patients with hematologic malignancies. PLoS One. 2019;14(12):e0225765. 31821331 10.1371/journal.pone.0225765 PMC6903763

[25] Jabbour JP , Ciotti G , Maestrini G , et al. Utility of procalcitonin and C‐reactive protein as predictors of gram‐negative bacteremia in febrile hematological outpatients. Support Care Cancer. 2022;30(5):4303‐4314. 35088150 10.1007/s00520-021-06782-w

[26] Schuetz P , Mueller B , Trampuz A . Serum procalcitonin for discrimination of blood contamination from bloodstream infection due to coagulase‐negative staphylococci. Infection. 2007;35(5):352‐355. 17882355 10.1007/s15010-007-7065-0

[27] Chaftari P , Chaftari AM , Hachem R , et al. The role of procalcitonin in identifying high‐risk cancer patients with febrile neutropenia: a useful alternative to the multinational association for supportive care in cancer score. Cancer Med. 2021;10(23):8475‐8482. 34725958 10.1002/cam4.4355 PMC8633259

[28] Munsell MK , Fadelu T , Stuver SO , et al. The utility of procalcitonin for diagnosing bacteremia and bacterial pneumonia in hospitalized oncology patients. J Cancer Res Clin Oncol. 2022:12. 10.1007/s00432-022-04419-x PMC11796829 36371720

[29] Schuetz P , Wirz Y , Sager R , et al. Procalcitonin to initiate or discontinue antibiotics in acute respiratory tract infections. Cochrane Database Syst Rev. 2017;10:CD007498. 29025194 10.1002/14651858.CD007498.pub3 PMC6485408

[30] Huang DT , Yealy DM , Angus DC , Pro ACTI . Longer‐term outcomes of the ProACT trial. N Engl J Med. 2020;382(5):485‐486. 31995699 10.1056/NEJMc1910508 PMC7332226

[31] Tujula B , Hamalainen S , Kokki H , Pulkki K , Kokki M . Review of clinical practice guidelines on the use of procalcitonin in infections. Infect Dis (Lond). 2020;52(4):227‐234. 31858869 10.1080/23744235.2019.1704860

[32] Shehabi Y , Seppelt I . Pro/con debate: is procalcitonin useful for guiding antibiotic decision making in critically ill patients? Crit Care. 2008;12(3):211. 18466649 10.1186/cc6860 PMC2481434

[33] Kamat IS , Ramachandran V , Eswaran H , Guffey D , Musher DM . Procalcitonin to distinguish viral from bacterial pneumonia: a systematic review and meta‐analysis. Clin Infect Dis. 2020;70(3):538‐542. 31241140 10.1093/cid/ciz545

[34] El Haddad H , Chaftari AM , Hachem R , Chaftari P , Raad II . Biomarkers of sepsis and bloodstream infections: the role of procalcitonin and Proadrenomedullin with emphasis in patients with cancer. Clin Infect Dis. 2018;67(6):971‐977. 29668936 10.1093/cid/ciy331

[35] Niu D , Huang Q , Yang F , et al. Serum biomarkers to differentiate Gram‐negative, Gram‐positive and fungal infection in febrile patients. J Med Microbiol. 2021;70(7):1‐8. 10.1099/jmm.0.001360 34259621

[36] Tao M , Zheng D , Liang X , He Q , Zhang W . Diagnostic value of procalcitonin for bacterial infections in patients undergoing hemodialysis: a systematic review and meta‐analysis. Ren Fail. 2022;44(1):81‐93. 35164633 10.1080/0886022X.2021.2021236 PMC8856046

[37] Bruno B , Busca A , Vallero S , et al. Current use and potential role of procalcitonin in the diagnostic work up and follow up of febrile neutropenia in hematological patients. Expert Rev Hematol. 2017;10(6):543‐550. 28471695 10.1080/17474086.2017.1326813

[38] Chaftari P , Qdaisat A , Chaftari AM , et al. Prognostic value of procalcitonin, C‐reactive protein, and lactate levels in emergency evaluation of cancer patients with suspected infection. Cancers (Basel). 2021;13(16):4087. 10.3390/cancers131674097 34439240 PMC8393196

[39] Wu CW , Wu JY , Chen CK , et al. Does procalcitonin, C‐reactive protein, or interleukin‐6 test have a role in the diagnosis of severe infection in patients with febrile neutropenia? A systematic review and meta‐analysis. Support Care Cancer. 2015;23(10):2863‐2872. 25701436 10.1007/s00520-015-2650-8

[40] Wudhikarn K , Palomba ML , Pennisi M , et al. Infection during the first year in patients treated with CD19 CAR T cells for diffuse large B cell lymphoma. Blood Cancer J. 2020;10(8):79. 32759935 10.1038/s41408-020-00346-7 PMC7405315

[41] Li S , Rong H , Guo Q , Chen Y , Zhang G , Yang J . Serum procalcitonin levels distinguish gram‐negative bacterial sepsis from Gram‐positive bacterial and fungal sepsis. J Res Med Sci. 2016;21:39. 27904585 10.4103/1735-1995.183996 PMC5122113

[42] Kloos WE , Bannerman TL . Update on clinical significance of coagulase‐negative staphylococci. Clin Microbiol Rev. 1994;7(1):117‐140. 8118787 10.1128/cmr.7.1.117 PMC358308

[43] Piukovics K , Terhes G , Lazar A , Timar F , Borbenyi Z , Urban E . Evaluation of bloodstream infections during chemotherapy‐induced febrile neutropenia in patients with malignant hematological diseases: single center experience. Eur J Microbiol Immunol. 2015;5(3):199‐204. 10.1556/1886.2015.00021 PMC4598887 26495130

[44] Schuetz P , Beishuizen A , Broyles M , et al. Procalcitonin (PCT)‐guided antibiotic stewardship: an international experts consensus on optimized clinical use. Clin Chem Lab Med. 2019;57(9):1308‐1318. 30721141 10.1515/cclm-2018-1181

[45] Schuetz P , Bolliger R , Merker M , et al. Procalcitonin‐guided antibiotic therapy algorithms for different types of acute respiratory infections based on previous trials. Expert Rev Anti Infect Ther. 2018;16(7):555‐564. 29969320 10.1080/14787210.2018.1496331

[46] Gregoriano C , Heilmann E , Molitor A , Schuetz P . Role of procalcitonin use in the management of sepsis. J Thorac Dis. 2020;12(Suppl 1):S5‐S15. 32148921 10.21037/jtd.2019.11.63 PMC7024752

[47] Xia Y , Zhang J , Li J , et al. Cytopenias following anti‐CD19 chimeric antigen receptor (CAR) T cell therapy: a systematic analysis for contributing factors. Ann Med. 2022;54(1):2951‐2965. 36382675 10.1080/07853890.2022.2136748 PMC9673810

[48] Evans L , Rhodes A , Alhazzani W , et al. Surviving sepsis campaign: international guidelines for Management of Sepsis and Septic Shock 2021. Crit Care Med. 2021;49(11):e1063‐e1143. 34605781 10.1097/CCM.0000000000005337

[49] Hill JA , Seo SK . How I prevent infections in patients receiving CD19‐targeted chimeric antigen receptor T cells for B‐cell malignancies. Blood. 2020;136(8):925‐935. 32582924 10.1182/blood.2019004000 PMC7441168

[50] Aitken SL , Nagel JL , Abbo L , et al. Antimicrobial stewardship in cancer patients: the time is now. J Natl Compr Canc Netw. 2019;17(7):772‐775. 31319394 10.6004/jnccn.2019.7318

